# Measles seroprevalence after reactive vaccination campaigns during the 2015 measles outbreak in four health zones of the former Katanga Province, Democratic Republic of Congo

**DOI:** 10.1186/s12889-019-7500-z

**Published:** 2019-08-22

**Authors:** Patrick Keating, Antonio Isidro Carrion Martin, Alexandre Blake, Pauline Lechevalier, Florent Uzzeni, Etienne Gignoux, Chibuzo Okonta, Céline Langendorf, Sheilagh Smit, Steve Ahuka, Melinda Suchard, Elizabeth Pukuta, Marie-Amélie Degail, Lisa Hansen, Jerry Kibanza-Kyungu, Iza Ciglenecki, Sandra Cohuet

**Affiliations:** 1 European Programme for Intervention Epidemiology Training, Stockholm, Sweden; 2 Epicentre, Paris, France; 3 Médecins Sans Frontières – Operational Center Paris, Paris, France; 4 Médecins Sans Frontières – Operational Center Geneva, Geneva, Switzerland; 5 National Institute for Communicable Diseases, Johannesburg, South Africa; 6 Institut National de Recherche Biomédicale, Kinshasa, Democratic Republic of Congo; 7 Division Provinciale Sanitaire du Tanganyika, Kalémie, Democratic Republic of Congo

**Keywords:** Measles, Seroprevalence, Immunity, Vaccination, Democratic Republic of Congo

## Abstract

**Background:**

Measles continues to circulate in the Democratic Republic of Congo, and the country suffered from several important outbreaks over the last 5 years. Despite a large outbreak starting in the former province of Katanga in 2010 and the resulting immunization activities, another outbreak occurred in 2015 in this same region. We conducted measles seroprevalence surveys in four health zones (HZ) in the former Katanga Province in order to assess the immunity against measles in children 6 months to 14 years after the 2015 outbreak.

**Methods:**

We conducted multi-stage cluster surveys stratified by age group in four HZs, Kayamba, Malemba-Nkulu, Fungurume, and Manono. The age groups were 6–11 months, 12–59 months, and 5–14 years in Kayamba and Malemba-Nkulu, 6–59 months and 5–14 years in Manono and Fungurume. The serological status was measured on dried capillary blood spots collected systematically along with vaccination status (including routine Extended Program of Immunization (EPI), and supplementary immunization activities (SIAs)) and previous self-reported history of suspected measles.

**Results:**

Overall seroprevalence against measles was 82.7% in Kayamba, 97.6% in Malemba-Nkulu, 83.2% in Manono, and 74.4% in Fungurume, and it increased with age in all HZs. It was 70.7 and 93.8% in children 12–59 months in Kayamba and Malemba-Nkulu, and 49.8 and 64.7% in children 6–59 months in Fungurume and Manono. The EPI coverage was low but varied across HZ. The accumulation of any type of vaccination against measles resulted in an overall vaccine coverage (VC) of at least 85% in children 12–59 months in Kayamba and Malemba-Nkulu, 86.1 and 74.8% in children 6–59 months in Fungurume and Manono. Previous measles infection in 2015-early 2016 was more frequently reported in children aged 12–59 months or 6–59 months (depending on the HZ).

**Conclusion:**

The measured seroprevalence was consistent with the events that occurred in these HZs over the past few years. Measles seroprevalence might prove a valuable source of information to help adjust the timing of future SIAs and prioritizing support to the EPI in this region as long as the VC does not reach a level high enough to efficiently prevent epidemic flare-ups.

**Electronic supplementary material:**

The online version of this article (10.1186/s12889-019-7500-z) contains supplementary material, which is available to authorized users.

## Background

A vaccine against measles, a highly contagious viral disease, has been available since 1963 and included in the World Health Organisation’s (WHO) Expanded Program on Immunisation (EPI) since 1974 [1, 2]. WHO and the United Nations International Children’s Emergency Fund (UNICEF) recommend that all children should receive two doses of the measles vaccine through routine immunisation services and/or through supplementary immunisation activities (SIAs) [3]. Global routine coverage with the first dose of the measles vaccine was estimated at 85% in 2015 [4]. Despite significant improvements in measles control over the past number of decades, there were still an estimated 109,638 measles deaths in 2017 [5].

The Democratic Republic of the Congo (DRC) implemented EPI in 1978, which includes the provision of one dose of the measles-containing vaccine (MCV) to infants aged 9–11 months [6]. The second dose is provided by regular SIAs among children aged 6–59 months. In 2010, the multiple indicator cluster survey (MICS) reported a national measles vaccine coverage (VC) of 72% among children aged 12–23 months, which is far below the 95% level required to prevent measles epidemics [7, 8]. A large measles epidemic took place in the DRC between 2010 and 2013 [9]. There were a total of 294,455 suspected or confirmed measles cases and approximately 5000 measles deaths notified in this period in the DRC, of which the majority of those affected were under 5 years of age [9]. The epidemic started in the former province of Katanga (in the south east of the DRC) and then spread across the north and north west of the country. The Ministry of Health (MoH) in collaboration with Médecins Sans Frontières (MSF) and UNICEF conducted more than 25 reactive vaccination campaigns across the country over the four-year period. Although high EPI vaccination coverage had been reported nationally, surveys carried out by MSF after the outbreak, found that the EPI VC ranged from 34 to 86% and VC due to the EPI or the reactive campaign ranged from 94 to 99% [6, 9].

Despite the great efforts employed to control the 2010–2013 epidemic and lower measles incidence in 2014, a measles epidemic was again declared in Katanga in early 2015 [10]. By November 2015, a total of 39,619 cases of measles, including 474 deaths, had been officially reported [11]. More than 77% of the children affected were aged 1–5 years. By early December, MSF teams had vaccinated almost 1 million children aged 6 months up to 14 years. Given the size of the epidemic in 2010–2013, the extensive immunisation activities in response to that epidemic and the reported high EPI VC levels, the reoccurrence of a measles epidemic in 2015 raised the question about the real measles immunity level among the population [6]. Antibody seroprevalence data can provide a more accurate estimate of the true immunity of a population against a given pathogen. The most recent measles seroprevalence data available for the DRC are from the 2013–2014 Demographic and Health Survey (DHS), which reported a national measles antibody seroprevalence of 64.4, and 51.4% in the former Katanga Province among children aged 6–59 months [12]. However, these estimates are of limited practical use at a more local level, due to the size of the former provinces and the likely spatial heterogeneity of seroprevalence.

**Fig. 1:**
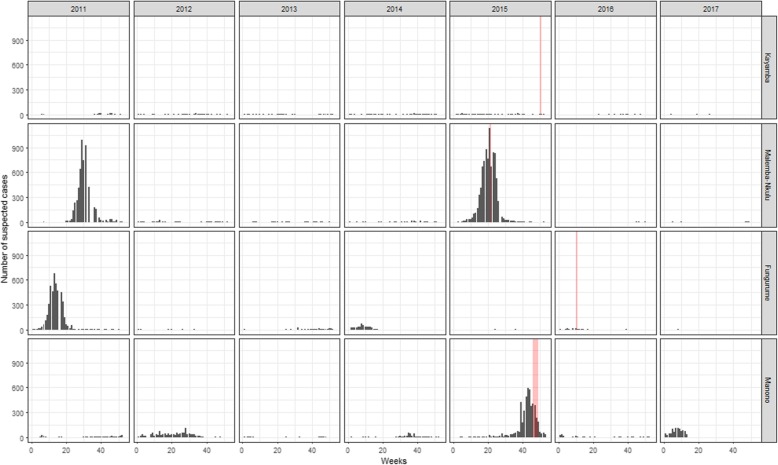
National surveillance data in Fungurume, Kayamba, Malemba-Nkulu and Manono from 2011-to 2017, with the vaccination campaigns in 2015 and 2016 indicated with a transparent ribbon

MSF provided support to the Ministry of Health (MoH) in case management and in the conduct of reactive vaccination campaigns across Katanga during the 2015 outbreak. Following the end of the outbreak, in early 2016, MSF and Epicentre conducted serosurveys in four health zones (HZ) of the former province of Katanga (Malemba Nkulu, Kayamba, Fungurume and Manono). These areas all benefitted from SIAs, and all but Fungurume also had a reactive vaccination campaign during 2015. Children 6–59 months could have benefitted an SIA in 2011, 2014, or early 2015 in Malemba-Nkulu, in 2011 and 2014 in Kayamba, in 2014 in Fungurume, and in 2012 in Manono. Additionally, they had been variably affected by the 2010–2013 and 2015 epidemics (Fig. 1), with attack rates (AR) during the latter in children under 5 of 132.0 cases/100000 persons in Kayamba, 2846.6 cases/100000 persons in Malemba-Nkulu, 1.4 cases/100000 persons in Fungurume, and 1778.9 cases/100000 persons in Manono (DRC official surveillance data, unpublished data). The objective of these serosurveys was to assess the level of seroprevalence in children aged 6 months to 14 years in these health zones.

## Methods

### Survey population

The study was conducted in four HZs of the former Katanga province, DRC: Malemba-Nkulu, Kayamba (now in Haut-Lomami Province), Manono (now Tanganyika Province), and Fungurume (now Lualaba Province), between 8th February and 25th March 2016. Because OCP and OCG had also specific questions in their respective surveys, the age strata varied between them. However, the age strata were done so that some comparison would still be possible across the four suveys. For Malemba-Nkulu and Kayamba we constructed three age strata: 6 to 11 months, 12 to 59 months and 5 to 14 years. In those same HZs, an additional stratification was done considering the presence of any cold chain material (fridges, or freezers) and the existence of a mobile vaccination team for routine vaccination at in health areas. The results of this stratification will not be presented because no relevant differences were found. For Manono and Fungurume, we used two age strata: 6 to 59 months and 5 to 14 years.

### Definitions

We defined a suspected measles case as an individual presenting with fever, non-vesicular maculopapular rash and at least one of the following signs: conjunctivitis, runny nose or cough.

### Sample size

Sample size was calculated for each of the four HZs assuming a 50% measles seroprevalence (given the absence of seroprevalence data in these specific areas), an α error of 5%, a desired precision of 10% and an anticipated design effect of 2. These calculations determined a minimum requirement of 193 children for each age strata.

### Sampling

We conducted a multi-stage cluster sample selection as described elsewhere [13]. For the first stage, we systematically sampled 20 clusters per HZ from the sampling frame proportionate to the respective population size of each village. The population estimates were obtained from each health zone data, as used for microplanification of vaccination campaigns and other public health interventions. In the second stage, we selected the first compound of households to sample using the EPI method [14]. Third, we randomly selected a single household among the compound households, and then interviewed and took samples from all eligible children. We then continued to the next (closest) compound until 10 children had been included for each age strata. If the last household interviewed had more children than the remaining number needed to complete the age strata, the number of children included was randomly chosen from the total number of children in that age strata within that household. In Malemba-Nkulu and Kayamba, the sampling was also stratified based on the availability of a fridge or freezer in the main health centre of the health area and the existence of a mobile vaccination team for routine vaccination. In total there were six strata in Malemba-Nkulu and Kayamba versus two in Fungurume and Manono. The results specific to the strata unrelated to age will not be presented.

### Data collection

Prior to data collection, all surveyors and supervisors underwent a three-day intensive training, including the practice of the data collection, sample collection, with simulations using several scenarios. With the support of MSF, data was collected in 5 days in Malemba-Nkulu and Kayamba, and in 3 to 4 days in Manono and Fungurume. Household heads were interviewed by teams of two trained interviewers, including at least one medical or paramedical staff member, using a standardised questionnaire on an electronic tablet (Additional file 1: Questionnaire). The questionnaire included information on the household and children. For the household, we collected information on health care access (presence of a nearby health centre (HC) and travel time required to reach it), and number of children within the study age group living in the household. For the children, we collected individual data on: age, sex, vaccination history (distinguishing between those verified with a card and an oral report) for routine, SIA, and reactive campaigns, and previous history of measles disease (distinguishing between those reported as being diagnosed at a health centre and those not diagnosed at a health centre). A calendar of events with vaccination periods of every SIA of the HZs during the last 15 years was used to help the parents remember when their children might have been vaccinated. Surveyors revisited the selected households after at least one adult came back from the field if any household was found locked during the first visit in rural areas.

### Sample collection and analysis

Trained interviewers collected capillary blood spots via fingerstick from all children for the IgG anti-measles measurement. We used a sterile lancet to obtain four drops (80 μL per drop) and placed them on Whatman 903, protein card savers. The samples were stored with silica gel desiccant using simple packaging and kept out of direct sunlight. Every sample was checked at the end of the day, and was stored using an additional packing. At the end of every survey, every sample was checked again and stored away from light using triple packing with silica gel desiccant at room temperature in a room with air conditioning. The samples were analysed at the National Institute for Communicable Diseases (NICD), South Africa using an indirect enzyme linked immunosorbent assay (ELISA) to measure anti -measles IgG with the Enzygnost commercial kit (Siemens). Two 6 mm disks were cut from each sample card and eluted overnight in 220 μL kit sample buffer at 4 °C. The manufacturer’s protocol was followed with the exception that 25 μL of the sample eluate was used. The optimum volume of eluate was determined using matched pairs of serum and DBS control samples in a separate pilot study (Additional file 2: Table S1). A titre less than 150 mUI/mL was reported as negative, a titre between 150 and 350 mIU/mL was considered equivocal, and titre higher than 350 mUI/mL was reported positive [15]. A cut-off point of 500 mIU/mL was used to define the clinical protection against the disease. A more conservative cut-off was selected to avoid overestimating the level of seroprevalence in the target population, for several reasons: i) there is no validated cut-off used as a surrogate to immunity with this technique in the literature, ii) this same cut-off has been used in a similar study [16], iii) it is necessary to use a higher cut-off than the one used with the gold standard plaque neutralization technique, (120 mIU/mL), given that the gold standard technique targets more specifically the functional neutralising IgG, while the ELISA used in this study detects IgG against measles and not specifically the neutralising IgG [15].

### Data entry and analysis

Data were directly entered by the interviewers on the tablets using the KoBoCollect application. The data management, cleaning and analysis were done using R 3.3.2 (The R Foundation for Statistical Computing).

The analysis was weighted to calculate averages, percentages, and their respective 95% confidence intervals taking into account the sampling design (the cluster and household levels) and the population estimations for each age strata. A sensitivity analysis was performed to assess if the interpretation of measles seroprevalence changed with variations of 100 mIU/mL below and above the chosen cut-off point.

## Results

We included 764 households and 3157 children in this study. The response rate was above 90%, refusals were met in only one cluster in Malemba-Nkulu and in one cluster in Manono. The number of children included, sex proportions and mean age by HZ and age group can be seen in Table 1. In Malemba-Nkulu, Kayamba, Fungurume, and Manono, the percentages of participants with a blood sample with valid results were 87.6, 88.3, 79.8, and 91.7% respectively.

**Table 1:** Distribution of children included, sex proportion and mean age by age group and health zone (Kayamba, Malemba-Nkulu, Fungurume, and Manono), former Katanga Province, Democratic Republic of Congo, 2016

Characteristics	Kayamba *N* = 1173			Malemba-Nkulu *N* = 1184			Fungurume *N* = 401		Manono *N* = 400	
	6–11 m *N* = 361	12–59 m *N* = 402	5–14 y *N* = 410	6–11 m *N* = 399	12–59 m *N* = 388	5–14 y *N* = 397	6–59 m *N* = 200	5–14 y *N* = 201	6–59 m N = 200	5–14 y *N* = 199
Sex of the children (%)										
Female	55.7	51.0	49.8	53.1	51.5	42.6	49.3	47.0	48.2	48.0
Male	44.3	49.0	50.2	46.9	48.5	57.4	50.7	53.0	51.8	52.0
Mean age	8.6^a^	36.1^a^	8.3^b^	9.1^a^	38.4^a^	9.0^b^	31.3^a^	8.7^b^	31.1^a^	8.7^b^

^a^Mean age in months

^b^Mean age in years

**Fig. 2:**
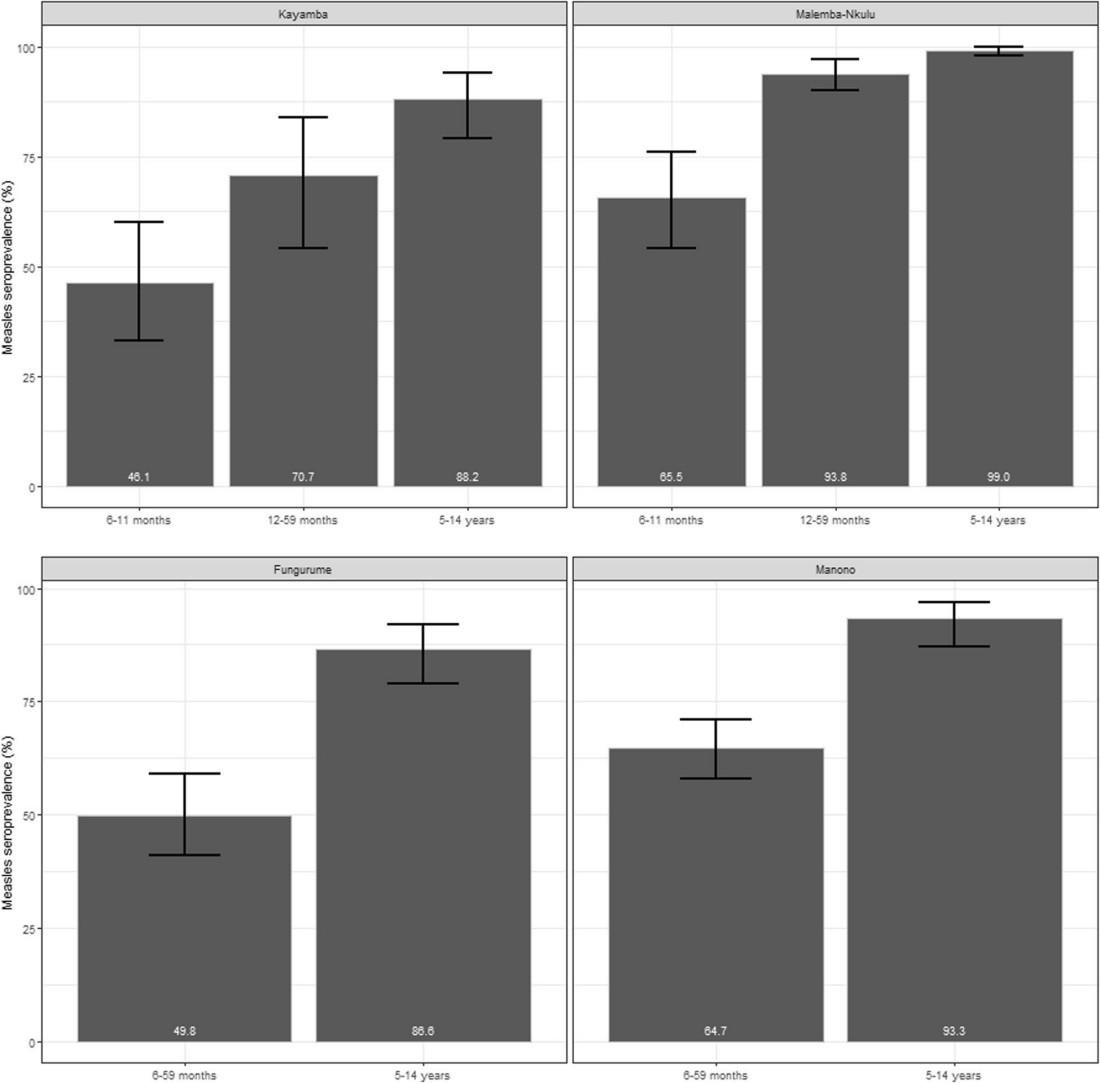
Measles seroprevalence among children aged 6 months to 14 years by age group in Kayamba, Malemba-Nkulu, Fungurume and Manono, DRC, 2016. EPI: Extended Programme on Immunization. SIA: Supplementary Immunization Activities. The number at the bottom of the bars indicate the coverage based on card or oral reporting

Overall measles seroprevalence (titre> 500 mIU/mL) was 82.7% (95% CI: 72.4–91.1) in Kayamba, 97.6% (95% CI:96.4–98.7) in Malemba-Nkulu, 83.2% (95% CI: 79.1–87.3) in Manono, and 74.4% (95%CI: 67.8–81.0) in Fungurume. It increased with increasing age in all HZs (Fig. 2). The lowest measles seroprevalance was found in Kayamba among children aged 6–11 months old (46.1, 95% CI: 33.0–60.0%) and the highest in Malemba Nkulu among children aged 5–14 years (99.0, 95% CI: 98.0–100%).

**Fig. 3:**
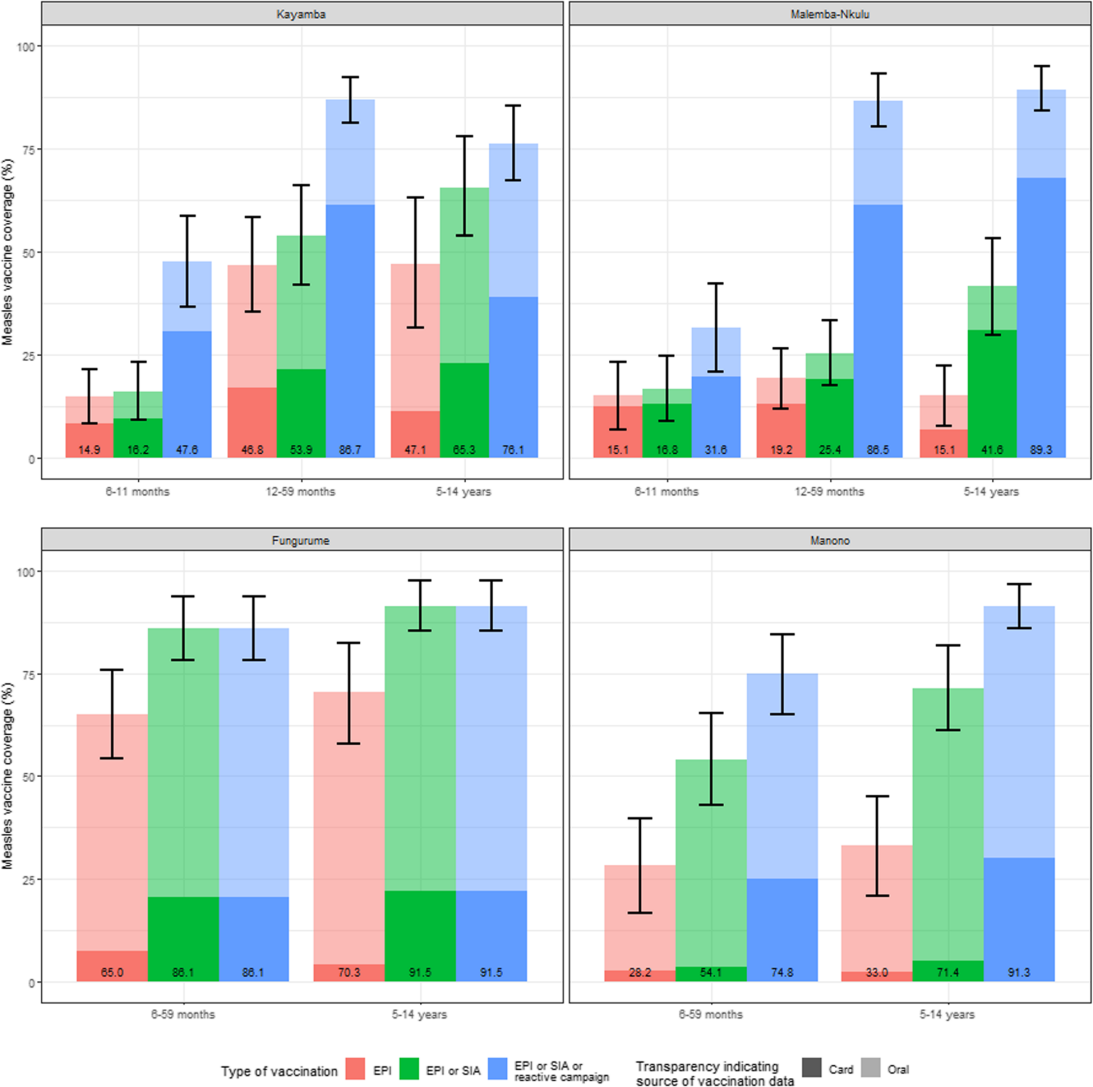
Vaccine coverage by age group, type of vaccination campaign and method of reporting in Kayamba, Malemba-Nkulu, Fungurume and Manono, DRC, 2016*. *Fungurume did not benefit from a recent reactive campaign before the survey. EPI: Extended Programme on Immunization. SIA: Supplementary Immunization Activities. The number at the bottom of the bars indicate the coverage based on card or oral reporting

The EPI coverage was low overall, but with important variations across HZs (Fig. 3). Confirmation of vaccination status was rarely possible through vaccination card (non-transparent color in Fig. 3), so the discrepancy between the VC based on card confirmation and the one based on card or self-reporting was important in all four HZs. In the rest of this article EPI coverage findings will refer to card or oral report (transparent color in Fig. 3). EPI coverage (red bars in Fig. 3) in Kayamba increased with age, from 14.9% in those aged 6–11 months to 47.1% to those aged 5–14 years. The EPI coverage in Malemba Nkulu was low and varied little by age group and peaked respectively at 19.2% in the first HZ and at 33.0% among 5–14 years old children in the latter The highest EPI coverage in any HZ was observed in Fungurume, with 65.0 and 70.3% of children aged 6–59 months and 5–14 years, respectively, receiving this routine dose.

The accumulation of the EPI, past SIAs, and the recent reactive vaccination campaigns (blue bars in Fig. 3) resulted in an overall VC of at least 85% in children 12–59 months in Kayamba and Malemba-Nkulu, 86.7 and 86.5% respectively. There was no reactive campaign in Fungurume, but the accumulation of EPI and SIA led to a VC of 86.1% in children 6–59 months, thanks to the highest EPI. Conversely, Manono reached a VC of 74.8% in children 6–59 months despite benefitting from a reactive campaign. In Kayamba and Malemba-Nkulu, the reactive campaigns enabled achievement of a VC as high as 85% with at least one dose in the older age groups (Fig. 3).

**Fig. 4:**
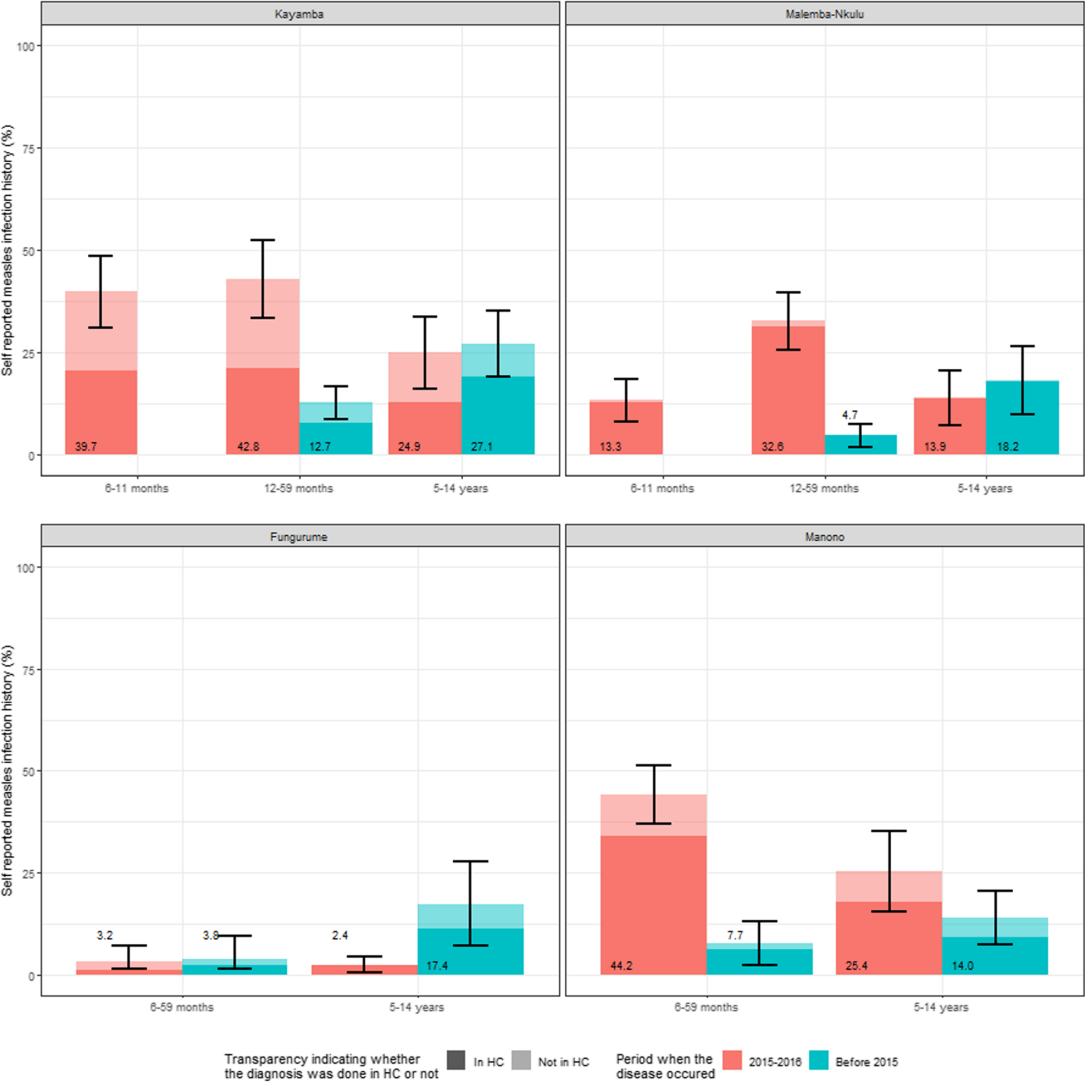
Past measles history by age group, time and place of diagnosis in Kayamba, Malemba-Nkulu, Fugurume, Manono, DRC, 2016. HC: Health Centre. EPI: Extended Programme on Immunization. SIA: Supplementary Immunization Activities. The number at the bottom of the bars indicate the coverage based on card or oral reporting

The children aged 12–59 months or 6–59 months (depending on the HZ) were the age groups amongst whom a previous measles infection in 2015-early 2016 was more frequently reported (Fig. 4). In Kayamba, 42.8% (95% CI: 33.2–52.3%) of the children aged 12–59 months reported a previous measles infection (diagnosed in a HC or not) and 32.6% (95% CI: 25.7–39.5%) in Malemba-Nkulu in the same age group. In Manono, 44.2% (95% CI: 37.1–51.2%) of the children aged 6–59 months reported a previous measles infection in 2015-early 2016 (diagnosed in a HC or not). In Fungurume, very few previous measles infections were reported in 2015-early 2016 (Fig. 4).

A sensitivity analysis showed that a variation of 100 mIU/mL around the chosen cut-off did not have a significant impact on the interpretation of the seroprevalence in the surveys (Additional file 3: Figure S1, Additional file 4: Figure S2, Additional file 5: Figure S3, Additional file 6: Figure S4).

## Discussion

The measles seroprevalence (titre> 500 mIU/mL) estimated by this study in the four target health zones was above 85% in children aged 5 years or older, but with considerable variation among younger age groups, depending on the HZ.

The level of protection is a consequence of either a natural infection or a successful vaccination. In this study, we estimated the level of protection, using seroprevalence as a proxy, by directly measuring antibody seroprevalence. The level of protection observed was largely consistent with the trends displayed by the AR in the national surveillance system (Fig. 1) and the VC achieved (Fig. 3). The seroprevalence (titre> 500 mIU/mL) in Kayamba in the three age groups (6 to 11 months: 46%, 12 to 59 months: 78%, and 5 to 14 years: 88%) was comparatively lower than Malemba-Nkulu (65, 94, and 99%, respectively) (Fig. 2). In Kayamba, few cases were reported, and a vaccination campaign was organised late by the MoH. Comparatively, Malemba-Nkulu reported the highest AR and benefitted from an MSF reactive vaccination campaign. In Manono, which also reported a high AR and benefitted from an MSF reactive vaccination campaign, the resulting seroprevalence was similar to that in Malemba-Nkulu, in the two age groups studied in this area (6 to 59 months: 65% and 5 to 14 years: 93%). Fungurume, which reported almost no cases and did not receive any reactive vaccination intervention, presented the lowest seroprevalence (6 to 59 months: 50%, and 5 to 14 years: 87%). Our findings are also consistent with the events that occurred in 2016 and 2017: Fungurume and Manono, with low seroprevalence in children under 5, had another measles outbreak in February 2016 and January 2017, respectively, and both subsequently benefited from a reactive campaign.

An important finding, and a likely cause of the 2015 outbreak, is the very low reported EPI coverage in Manono, Malemba-Nkulu and Kayamba (Fig. 3). We should take into account that the minimum age for measles-containing vaccine (MCV) in the DRC EPI is 9 months, but we included children over the age of 6 months in our study [6]. Therefore, this could partially explain the low coverage in the younger group. Our estimates of the EPI coverage were substantially lower than the administrative coverages used locally (unpublished data) to monitor their activity. This could be explained by the inaccuracy of the denominator used, in the absence of a recent census. Nonetheless, these low coverage estimates are consistent with the proportions of children reporting prior measles infection in 2015–2016 in these three HZs (Fig. 4). The high AR in Manono and Malemba-Nkulu, as estimated from the national surveillance notifications, is also consistent with the low EPI coverage that we found in those two areas. Interestingly, the number of cases reported by the surveillance system in Kayamba is not consistent with the percentage of children reporting prior measles infection in our survey; this could be due to underreporting in the surveillance system, or by over-reporting of prior measles infection in our survey since about half of those past measles infections had not been checked in a HC. Conversely, the highest reported EPI coverage among the four HZs was found in Fungurume, where very few cases were notified, and no reactive vaccination took place in the HZ. This is consistent with the literature identifying that improving the EPI is the most cost effective long–term option to increase the protection of the population [17].

Regarding the additional coverage offered by each of the three vaccination types, the importance of SIA and reactive campaigns on an otherwise very low VC can be clearly appreciated in Fig. 3. Particularly interesting is the great boost that reactive vaccination adds to the overall VC, especially in children aged 12–59 months in Kayamba and Malemba-Nkulu, children who were old enough to be targeted by EPI and SIA and also by reactive campaign. Besides, except in Fungurume, the reactive campaigns enabled achievement of a VC at least as high as 85% in Kayamba and Malemba-Nkulu. The EPI and the SIA were not enough to provide a VC, which would prevent an outbreak in those two HZs, and regular flare-ups are therefore bound to happen.

These are the first surveys to gather seroprevalence data in DRC at the HZ scale, which is small enough to guide interventions. The 2013–2014 DHS reported seroprevalence data in DRC, but at the level of the former Provinces, some of which were as big as Spain. Besides, the high fertility in this region [12] could increase the pool of susceptible within a short period of time. Serosurveys can provide a better estimate of the true immunity of a population against a pathogen, but they are costly to implement. WHO (European region) recommends the use of serosurveys, as part of a measles elimination strategy, in countries where surveillance and vaccine coverage data are unreliable [18]. In addition, a recent simulation study of measles incidence in a variety of settings, suggested that small scale serosurveys have the potential to serve as an additional tool for measles control [19]. In contexts like the DRC, vaccination campaigns triggered by serosurveys could have a great impact on averting measles cases. However, few countries are currently utilising this strategy.

There are several limitations to these surveys. First due to logistical constraints, we used the classic EPI method of spinning the pen for selection of the first household, as time constraints on implementation did not permit a probability-based sample, as recommended by the updated WHO reference manual on vaccination coverage cluster surveys due to accessibility constraints [20, 21]. This could have created some bias in household selection, in that houses closer to the centre of a village were more likely to be selected. Second, considering the challenge of taking blood samples on approximately 3000 children in remote areas, seroprevalence was not measured with the reference technique, the PNRT, but with an ELISA, the Enzygnost commercial kit (Siemens©) and not on serum but on dried blood spots (DBS). This complicates the interpretation of the IgG titre since there is no clear surrogate to clinical protection with ELISA techniques and various studies have used different cut-offs [15, 22]. Because of the lower sensitivity due to the technique and the DBS, the wish not to overestimate the protection in a country prone to measles outbreaks, and based on similar studies, we used a cut-off of 500 mIU/mL to define clinical protection [16]. In any case, the sensitivity analysis showed no substantial impact of a reasonable variation of the chosen cut-off on the interpretation of our findings (Additional file 3: Figure S1, Additional file 4: Figure S2, Additional file 5: Figure S3, Additional file 6: Figure S4). Third, although antibody is highly correlated with protection, it is still not a perfect correlate of protection as it doesn’t measure cellular immunity and those seronegative and vaccinated might be still protected [23]. In addition, logistical constraints delayed the shipment of the samples and they reached the NICD laboratory 3 months after the end of the data collection. Despite storing the samples in good conditions this could lead to an underestimation of the seroprevalence. Finally, the infection and vaccination history were mostly self-reported since very few vaccination cards were found, and we could not cross check our data with local health centre registers. It is difficult to distinguish one eruptive disease from another, and parental recall as children age introduced an inevitable memory bias (i.e. the older the children the more difficult to recall). The weak reliability of self-reported data on measles vaccination has already been described, and although it makes the interpretation more complex, it is an additional rationale for the use of seroprevalence surveys in situations where health records are poor [24].

## Conclusions

In conclusion, the conjunction of all these factors: i) varying measles seroprevalence, ii) low EPI vaccination coverage in children under 5 years, iii) high fertility rates and iv) the average 3 years gap between SIAs with suboptimal coverage, creates conditions conducive for future outbreaks. In fact, only 3 to 6 months after 23 reactive vaccination campaigns in this region for this outbreak (realized by MSF or other partners), we already observed in the seroprevalence data a low protection of children under 5 years. Hence guiding and supporting immunisation activities in places most at risk, such as the DRC, is of utmost priority. In this regard, our findings on low routine EPI coverage might support the introduction of a second dose of MCV in the routine schedule [25, 26]. Improving the EPI coverage, the targeting, timeliness, and also the coverage of SIAs are key to reduce the epidemic risk. Where reliable surveillance and vaccine coverage data are not available, serosurveys could serve as an additional measles elimination tool through guiding decision making on the need for/timing of SIAs. Furthermore, they might provide valuable information for risk assessment and epidemic preparedness in regions with sustained measles circulation where vaccination coverage is not expected to improve in the short term.

## Additional files

Additional file 1: questionnaire. (DOCX 21 kb)

Additional file 2: **Table S1.** Results of the pilot study realized by the National Institute for Communicable Diseases showing the test results of sera from 11 volunteers and their matched dried blood spots samples collected on protein saver cards using different dilutions and elution buffers. ^†^DBS: Dried Blood Spots. *OD: Corrected optical density. ^PBS-TM: Phosphate-buffered saline containing 0.5% (v/v) Tween-20 and 5% (m/v) non-fat milk powder. OD < 0.1 was considered negative, 0.1 ≤ OD < 0.2 was considered equivocal, and OD ≥ 0.2 was considered positive. (DOCX 28 kb)

Additional file 3: **Figure S1.** Impact of varying the IgG threshold value on the proportion of protected children by age group in Kayamba (the red vertical line is the threshold used). The grey area corresponds to the threshold used (500 mIU/mL) +/− 100 mIU/mL. (TIFF 1054 kb)

Additional file 4: **Figure S2.** Impact of varying the IgG threshold value on the proportion of protected children by age group in Malemba-Nkulu (the red vertical line is the threshold used). The grey area corresponds to the threshold used (500 mIU/mL) +/− 100 mIU/mL. (TIFF 1054 kb)

Additional file 5: **Figure S3.** Impact of varying the IgG threshold value on the proportion of protected children by age group in Fungurume (the red vertical line is the threshold used). The grey area corresponds to the threshold used (500 mIU/mL) +/− 100 mIU/mL. (TIFF 1054 kb)

Additional file 6: **Figure S4.** Impact of varying the IgG threshold value on the proportion of protected children by age group in Manono (the red vertical line is the threshold used). The grey area corresponds to the threshold used (500 mIU/mL) +/− 100 mIU/mL. (TIFF 1054 kb)

## Data Availability

The datasets generated and analysed during the current study are available in the Open Science Framework repository, https://osf.io/978er/.

## Abbreviations

AR: Attack Rate
DBS: Dried blood spots
DHS: Demographic and Health Survey
DRC: Democratic Republic of Congo
ELISA: Enzyme Linked Immunosorbent Assay
EPI: Extended Program of Immunization
HZ: Health zone
MCV: Measles-Containing Vaccine
MoH: Ministry of Health
MSF: Médecins Sans Frontières
NICD: National Institute for Communicable Diseases
PNRT: Plaque Neutralisation Reduction Test
SIAs: Supplementary Immunization Activities
UNICEF: United Nations International Children’s Emergency Fund
VC: Vaccine coverage
WHO: World Health Organisation
