# Supplementary material for: Measles seroprevalence after reactive vaccination campaigns during the 2015 measles outbreak in four health zones of the former Katanga Province, Democratic Republic of Congo
Source: BMC Public Health. 2019 Aug 22;19:1153. doi: 10.1186/s12889-019-7500-z (PMC6704676; doi:10.1186/s12889-019-7500-z)
Supplement: Supplementary file 1 — questionnaire. (DOCX 21 kb) [file 12889_2019_7500_MOESM1_ESM.docx]

| **Variable** | **Coding** |
| --- | --- |
| Country | Text (pre-filled) |
| Province | Text (pre-filled) |
| Health Zone | Text (pre-filled) |
| Health Area | Text (predefined list) |
| Group number | Number between 1 and 10, predefined list |
| Village | Automatic based on group list |
| Date of study | Automatic (by default the day that the questionnaire is filled out) |
| Household ID | Automatic by group |
| Number of children in the household, 6 to 59 months old | Number |
| Number of children in the household, 5 to 14 years old | Number |
| Consent | Y/N |
| Geographical and medical accessibility |  |
| Presence of a main road nearby that can be negotiated by care or motorcycle? | Y/N |
| Presence of a functional runway nearby? | Y/N |
| Presence of a HC or HP nearby? | Y/N |
| Presence of a road linking the HC to the BCZS that can be negotiated by car or motorcycle? | Y/N |
| Transit time to the nearest HC/HP? | Number between 1 and 24 in hours OR  Number between 1 and 7 in days |
| Presence of healthcare personnel at the nearest HC/HP? | Y/N |
| Repeat the following questions for each child between 6 and 59 months old |  |
| Child ID | Automatic by group |
| Age (in months) if the child is 6 to 59 months old | Between 6 and 59 |
| Age (in years) if the child is 5 to 14 years old | Between 5 and 14 |
| Gender | Girl or boy |
| EPI vaccination with card | Y/N |
| Number of PEV doses | Number between 1 and 2 |
| Part of the body where the vaccine was administered | Right or left |
| SIA vaccination with card | Y/N |
| Part of the body where the vaccine was administered | Right or left |
| Number of SIA doses | Number between 1 and 2 |
| Declared vaccination (without card) | Y/N |
| Part of the body where the vaccine was administered | Right or left |
| Number of declared doses received | 0, 1 or 2, expect “don’t know” |
| Verification of the number of total doses received with card | EPI + SIA (if incorrect, go back to the previous questions) |
| Verification of the number of total doses with or without card | EPI + SIA + without card (if incorrect, go back to the previous questions) |
| Has the child had a measles virus infection in the last 6 months? | Y/N (see definition of a case) |
| If so, when? | Month/year |
| Diagnosed at the HC? | Y/N |
