# Supplementary material for: Measles seroprevalence after reactive vaccination campaigns during the 2015 measles outbreak in four health zones of the former Katanga Province, Democratic Republic of Congo
Source: BMC Public Health. 2019 Aug 22;19:1153. doi: 10.1186/s12889-019-7500-z (PMC6704676; doi:10.1186/s12889-019-7500-z)
Supplement: Supplementary file 2 — Table S1. Results of the pilot study realized by the National Institute for Communicable Diseases showing the test results of sera from 11 volunteers and their matched dried blood spots samples collected on protein saver cards using different dilutions and elution buffers. †DBS: Dried Blood Spots. *OD: Corrected optical density. ^PBS-TM: Phosphate-buffered saline containing 0.5% (v/v) Tween-20 and 5% (m/v) non-fat milk powder. OD < 0.1 was considered negative, 0.1 ≤ OD < 0.2 was considered equivocal, and OD ≥ 0.2 was considered positive. (DOCX 28 kb) [file 12889_2019_7500_MOESM2_ESM.docx]

Additional file 2: Table S1. Results of the pilot study realized by the National Institute for Communicable Diseases showing the test results of sera from 11 volunteers and their matched dried blood spots samples collected on protein saver cards using different dilutions and elution buffers.

| Participant ID | Serum | |  | DBS^†^ eluted in 15µl PBS-TM^ | |  | DBS^†^ eluted in 20µl PBS-TM^ | |  | DBS^†^ eluted in 25µl PBS-TM^ | |  | DBS^†^ eluted in 25µl sample buffer | |
| --- | --- | --- | --- | --- | --- | --- | --- | --- | --- | --- | --- | --- | --- | --- |
|  | OD* | Interpretation |  | OD* | Interpretation |  | OD* | Interpretation |  | OD* | Interpretation |  | OD* | Interpretation |
| 1 | 0.289 | Positive |  | 0.18 | Equivocal |  | 0.22 | Positive |  | 0.26 | Positive |  | 0.28 | Positive |
| 2 | 1.415 | Positive |  | 0.97 | Positive |  | 1.52 | Positive |  | 1.15 | Positive |  | 1.32 | Positive |
| 3 | 1.925 | Positive |  | 1.61 | Positive |  | 1.6 | Positive |  | 1.73 | Positive |  | 1.45 | Positive |
| 4 | 0.11 | Equivocal |  | 0.1 | Equivocal |  | 0.13 | Equivocal |  | 0.11 | Equivocal |  | 0.2 | Positive |
| 5 | 0.78 | Positive |  | 0.13 | Equivocal |  | 0.7 | Positive |  | 0.58 | Positive |  | 0.72 | Positive |
| 6 | 0.45 | Positive |  | 0.31 | Positive |  | 0.4 | Positive |  | 0.36 | Positive |  | 0.41 | Positive |
| 7 | 1.255 | Positive |  | 0.8 | Positive |  | 0.92 | Positive |  | 1.01 | Positive |  | 0.9 | Positive |
| 8 | 0.345 | Positive |  | 0.26 | Positive |  | 0.27 | Positive |  | 0.27 | Positive |  | 0.35 | Positive |
| 9 | 0.325 | Positive |  | 0.19 | Equivocal |  | 0.25 | Positive |  | 0.23 | Positive |  | 0.26 | Positive |
| 10 | 0.935 | Positive |  | 0.66 | Positive |  | 0.86 | Positive |  | 0.8 | Positive |  | 0.75 | Positive |
| 11 | 0.225 | Positive |  | 0.15 | Equivocal |  | 0.19 | Equivocal |  | 0.19 | Equivocal |  | 0.21 | Positive |

^†^DBS: Dried Blood Spots

*OD: Corrected optical density

^PBS-TM: Phosphate-buffered saline containing 0.5% (v/v) Tween-20 and 5% (m/v) non-fat milk powder

OD<0.1 was considered negative, 0.1≤OD<0.2 was considered equivocal, and OD≥0.2 was considered positive.

A previous study (1) showed that dried venous blood (DVB) samples eluted with kit sample buffer showed significantly better linear correlation to the serum sample than did DVB samples eluted with PBS-TM. NICD results showed a similar linear correlation of DBS eluted in PBS-TM and kit sample buffer but the percentage difference between serum and DBS results was smaller (i.e. better) using the kit sample buffer despite the high background. Furthermore, the sample buffer is provided with the kit and therefore no additional reagents need to be prepared. The NICD thus chose to use the kit sample buffer as the DBS elution buffer and to use 25 µl aliquots for testing as this closely approximates results using serum samples.

References:

1. Riddell MA, Byrnes GB, Leydon JA, Kelly HA. Dried venous blood samples for the detection and quantification of measles IgG using a commercial enzyme immunoassay. Bull World Health Organ. 2003;81(10):701–7.
