# Supplementary figures and images for: Measles seroprevalence after reactive vaccination campaigns during the 2015 measles outbreak in four health zones of the former Katanga Province, Democratic Republic of Congo
Source: BMC Public Health. 2019 Aug 22;19:1153. doi: 10.1186/s12889-019-7500-z (PMC6704676; doi:10.1186/s12889-019-7500-z)

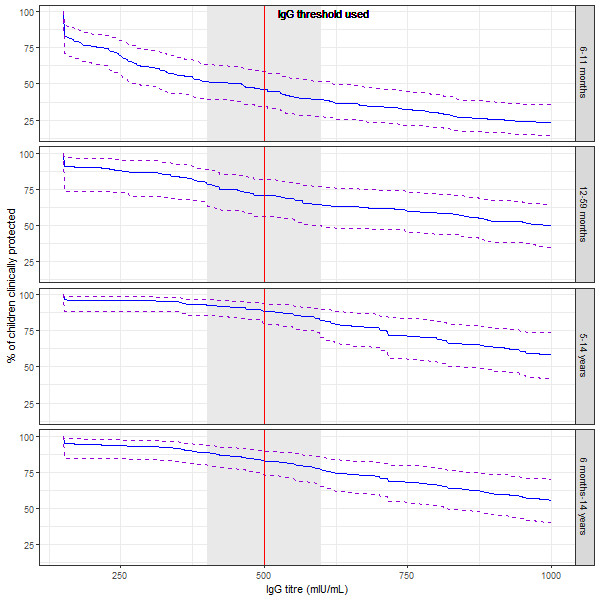

Supplement: Supplementary file 3 — Figure S1. Impact of varying the IgG threshold value on the proportion of protected children by age group in Kayamba (the red vertical line is the threshold used). The grey area corresponds to the threshold used (500 mIU/mL) +/− 100 mIU/mL. (TIFF 1054 kb) [file 12889_2019_7500_MOESM3_ESM.tiff]

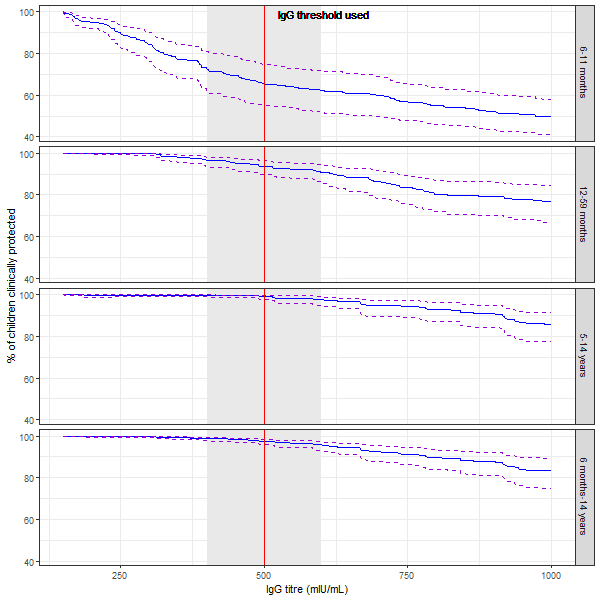

Supplement: Supplementary file 4 — Figure S2. Impact of varying the IgG threshold value on the proportion of protected children by age group in Malemba-Nkulu (the red vertical line is the threshold used). The grey area corresponds to the threshold used (500 mIU/mL) +/− 100 mIU/mL. (TIFF 1054 kb) [file 12889_2019_7500_MOESM4_ESM.tiff]

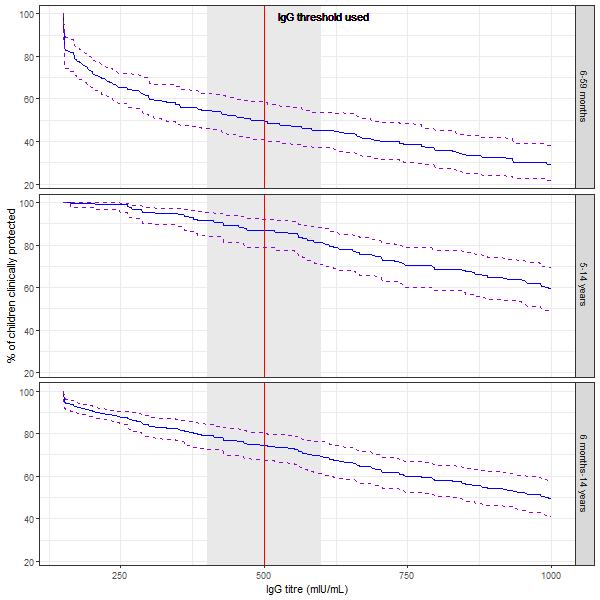

Supplement: Supplementary file 5 — Figure S3. Impact of varying the IgG threshold value on the proportion of protected children by age group in Fungurume (the red vertical line is the threshold used). The grey area corresponds to the threshold used (500 mIU/mL) +/− 100 mIU/mL. (TIFF 1054 kb) [file 12889_2019_7500_MOESM5_ESM.tiff]

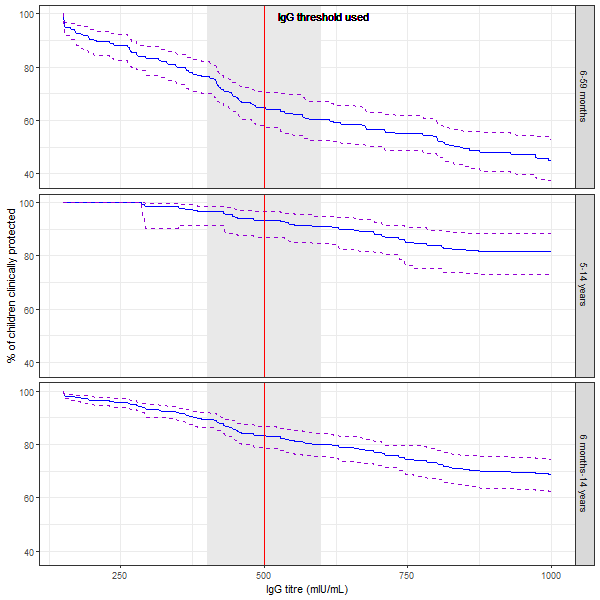

Supplement: Supplementary file 6 — Figure S4. Impact of varying the IgG threshold value on the proportion of protected children by age group in Manono (the red vertical line is the threshold used). The grey area corresponds to the threshold used (500 mIU/mL) +/− 100 mIU/mL. (TIFF 1054 kb) [file 12889_2019_7500_MOESM6_ESM.tiff]
